# Robot-assisted laparoscopic total gastrectomy for gastric cancer with common hepatic artery passed behind the portal vein: A case report

**DOI:** 10.1016/j.ijscr.2023.108561

**Published:** 2023-07-21

**Authors:** Yuto Sakurai, Yuma Ebihara, Yo Kurashima, Soichi Murakami, Toshiaki Shichinohe, Satoshi Hirano

**Affiliations:** Department of Gastroenterological Surgery II, Hokkaido University Faculty of Medicine, North 15 West 7, Kita-ku, Sapporo 0608638, Hokkaido, Japan

**Keywords:** Gastric cancer, Robotic gastrectomy, Conversion surgery, Adachi's classification

## Abstract

**Introduction:**

It is essential to identify variations of celiac artery (CA) and common hepatic artery (CHA), using preoperative computed tomography (CT) imaging, for safe gastrectomy and lymph node dissection in gastric cancer (GC) surgery. We report a relatively rare case with the CHA passing behind the portal vein (PV), in which we performed robot-assisted total gastrectomy (RTG) after chemotherapy as conversion surgery.

**Case presentation:**

A 78-year-old man with GC was referred for conversion surgery. Three-dimensional CT angiography revealed an anomalous CHA passing behind the PV. The anomaly corresponded to type I according to Adachi's classification, and the patient underwent robot-assisted laparoscopic total gastrectomy D2 lymphadenectomy (RTG D2) with Roux-en-Y reconstruction. The operation time was 543 min, blood loss was 115 ml, and no intraoperative complications occurred. The postoperative course was uneventful.

**Clinical discussion:**

A word of caution during the surgical procedure entails the manipulation of the suprapancreatic lymph node dissection. Initially, it is crucial to identify the anterior surface of the portal vein (PV) and the nerve plexus surrounding the common hepatic artery (CHA). After completely dissecting the entire circumference, the PV is secured using vascular tape. By gently pulling the vascular tape towards the ventral aspect, a safe execution of lymph node dissection no.8 and 12 on the dorsal side of the PV can be accomplished. Meticulous handling of the anatomical abnormalities observed in the preoperative images may prevent unintended hemorrhage.

**Conclusion:**

We report a case with vascular anomalies in which RTG D2 was performed successfully as a conversion surgery.

## Highlights

• Robotic surgery for gastric cancer is on the rise.
• Robot-assisted gastrectomy is feasible for performing lymphadenectomy safely in conversion surgery with vascular anomalies.
• Preoperative enhanced computed tomography imaging is necessary to identify the vascular abnormality.

## 1 Introduction

Gastric cancer is one of the most common malignancies and remains the 5th leading cause of cancer-related deaths worldwide [1]. The standard surgery for patients with resectable gastric cancers is gastrectomy with lymph node dissection. With advances in chemotherapy, including anticancer and molecular-targeted drugs, conversion surgery has become increasingly valuable, with improvements in long-term prognosis. It is essential to identify variations of celiac artery (CA) and common hepatic artery (CHA) using preoperative computed tomography (CT) imaging for safe gastric cancer (GC) surgery. There is a report of laparoscopic surgery for a gastric cancer patient with a rare vascular anatomical anomaly of Adachi type VI (group26), which is noted to frequency of 0.4 %, and even there the importance of preoperative imaging evaluation is emphasized [2]. Here, we report a rare vascular anomaly with common hepatic artery passed behind the portal vein with GC, who underwent robot-assisted laparoscopic total gastrectomy D2 lymphadenectomy (RTG D2) as conversion surgery. The case report adheres to SCARE criteria [3].

## 2 Presentation of case

**Fig. 1:**
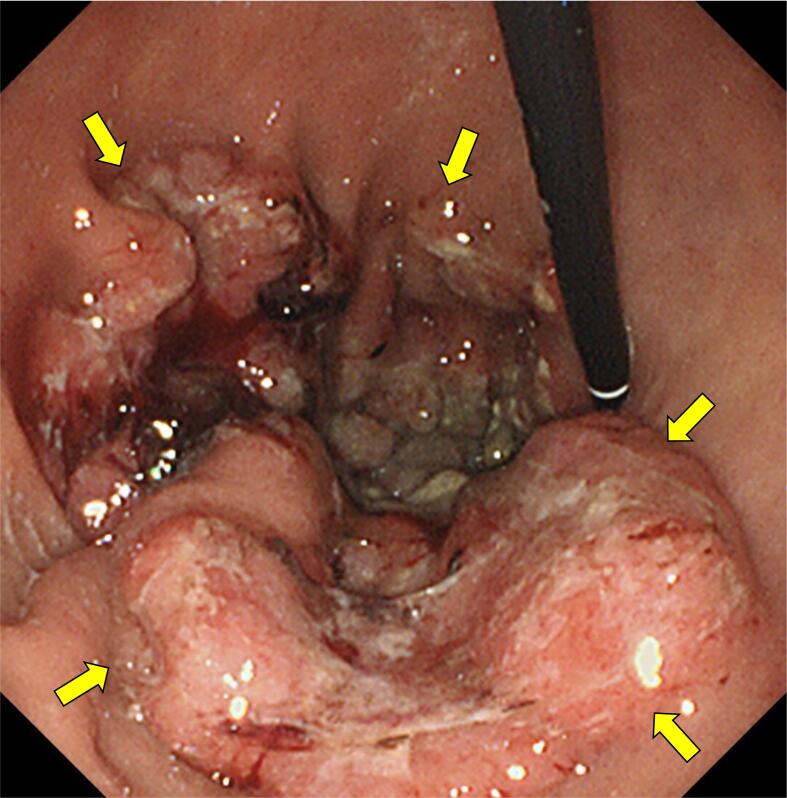
Esophagogastroduodenoscopy showing a type 3 lesion from the cardia to the greater curvature of the gastric body region.

**Fig. 2:**
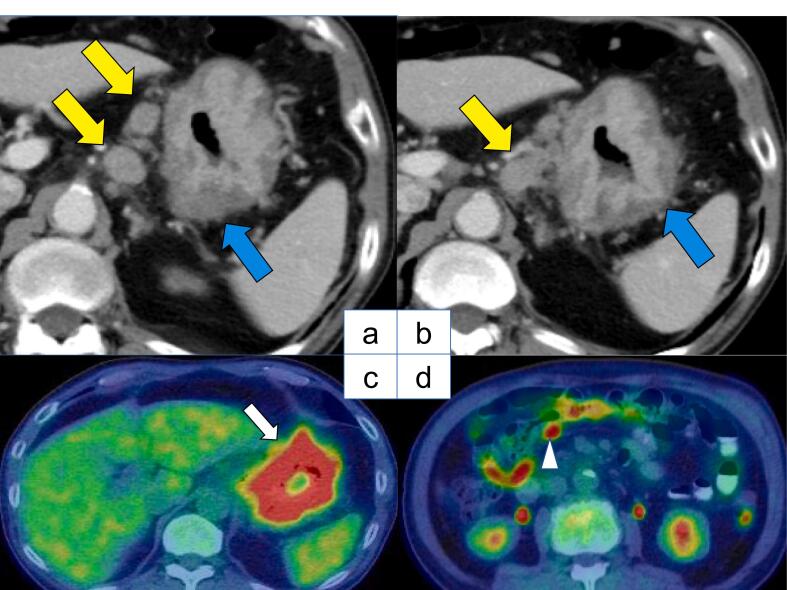
a, b - Images of pretreatment abdominal computed tomography (CT). The blue arrow shows irregular gastric wall thickening on esophagogastroduodenoscopy, consistent with a neoplastic lesion. The yellow arrow shows the enlarged lymph nodes of the lesser curvature. c - Images of pretreatment positron emission tomography (PET)–CT images. The white arrow shows uptake along the cardia to the greater curvature of the gastric region. d - The white arrowhead shows the peritoneal nodule demonstrating high fluorodeoxyglucose (FDG) uptake. (For interpretation of the references to color in this figure legend, the reader is referred to the web version of this article.)

**Fig. 3:**
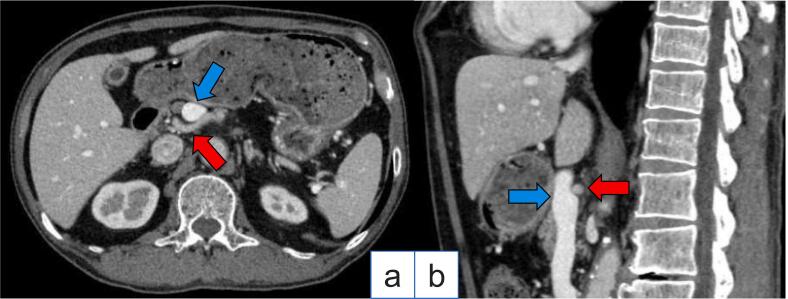
Images of preoperative abdominal computed tomography (CT). a - Axial view. Vascular anomalies with common hepatic artery (the red arrow) passing behind the portal vein (the blue arrow). b - Sagittal view. (For interpretation of the references to color in this figure legend, the reader is referred to the web version of this article.)

**Fig. 4:**
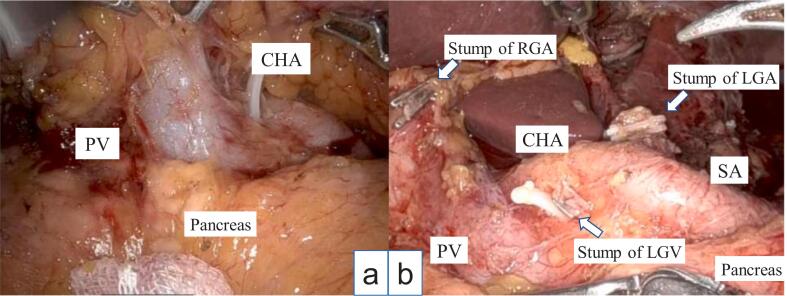
Intraoperative images. Vascular anomaly with the common hepatic artery passing behind the portal vein (Adachi's classification type I). a - Before lymph node dissection. b - After adequate lymph node dissection (D2). CHA, common hepatic artery; PV, portal vein; SA, splenic artery; RGA, right gastric artery; LGA, left gastric artery; LGV, left gastric vein.

A 78-year-old Japanese man visited our hospital with the complaint of upper abdominal pain after eating. Esophagogastroduodenoscopy revealed a type 3 lesion extending from the cardia to the greater curvature of the gastric body region (Fig. 1). GC was diagnosed after biopsy revealed a moderately-poorly differentiated adenocarcinoma. Abdominal CT showed suspected regional lymph node metastases (Fig. 2a, b), and the peritoneal nodule showed high fluorodeoxyglucose (FDG) uptake on positron emission tomography (PET) (Fig. 2c, d). Based on the findings, the patient was diagnosed as GC, T4aN3aM1 cStage IVB (UICC TNM 8th edition). Chemotherapy was initiated for the unresectable GC - oxaliplatin/5-fluorouracil/leucovorin (mFOLFOX) was administered for six courses followed by two courses of capecitabine/oxaliplatin (CapeOX) + trastuzumab. Abdominal CT showed partial response (PR) according to the Response Evaluation Criteria in Solid Tumors (RECIST) guidelines, and in the absence of disseminated disease, a radical resection could be considered; therefore, we decided to perform a staging laparoscopy (SL). SL showed no peritoneal nodules or liver metastases, and the patient was diagnosed with ycT4aN3aM0 ycStage III (UICC TNM 8th edition). After SL, the patient underwent RTGD2 with Roux-en-Y reconstruction as conversion surgery, after the third course of CapeOX + trastuzumab. The operation was performed under general anesthesia with lithotomy. Six ports were used as previously reported [4], and all gastrointestinal anastomoses were performed by intraperitoneal manipulation. A vascular anomaly was seen in this case, with the CHA passing behind the portal vein (PV) (Fig. 3). CA was classified as Adachi's type I, which is the most typical pattern [5]. Adequate lymph node dissection was performed while avoiding vascular injury (Fig. 4). The operation time was 543 min, there was 115 ml of blood loss, and no intraoperative complications occurred. The postoperative course was uneventful - the patient resumed drinking water and eating on postoperative days 1 and 3, respectively, and was discharged on postoperative day 14. The lymph node regions and dissection were defined according to the Japanese Classification of Gastric Carcinoma [6]. The pathological findings showed tubular adenocarcinoma with invasion of the subserosa, and one regional lymph node metastasis. Consequently, the patient was diagnosed as GC ypT3N1M0 ypStage IIB (UICC TNM 8th edition). After obtaining informed consent, adjuvant therapy was administered, and the patient had no recurrence 8 months postoperatively.

## 3 Discussion

Robot-assisted surgery for GC was first reported in 2003 [7]. It provides high-resolution three-dimensional (3D) images, use of forceps with multi-joint functions and handshake prevention, thus eliminating the limitations of conventional laparoscopic gastrectomy. GC surgery is considered a favorable indication for robot-assisted surgery, as it requires procedures for anatomically sophisticated structures and elaborates lymph node dissection for suprapancreatic lymphadenectomy [8]. Although several studies have compared robot-assisted distal gastrectomy (RDG) to laparoscopic distal gastrectomy (LDG) [9,10], there remains a lack of agreement on the safety and efficacy of robot-assisted surgery for GC. Short-term clinical outcomes of recently published trials on the usefulness of robot-assisted surgery for GC observed lesser intraoperative blood loss, accurate lymph node dissection, improved postoperative recovery, lesser postoperative complication rate, and longer time to postoperative adjuvant chemotherapy induction, than after LDG [11]. Further reports comparing long-term outcomes, including disease-free survival, are awaited.

In GC surgery, it is essential to preoperatively identify the branching artery of the CA to ensure lymph node dissection. There are various presentations of CA branches, divided into 28 groups of 6 types by Adachi's classification [5][. In the present case, CT showed that the CHA originated from the CA, which is categorized as Adachi classification type I, but focusing on the relationship with the PV, CHA passed behind the PV was observed as an anomaly. In previous studies, vascular anomalies have been reported in 0.1–0.12 % of all cases [[13], [14], [15], [16]]. In this case, there is no evidence of hepatic artery anomaly, but it has been reported that the right hepatic artery passed behind the PV when the right hepatic artery branches from the CHA proximally from the left margin of the PV or when the right hepatic artery branches from the superior mesenteric artery [17]. Wadhwa reported a case that is the abnormal rotation of midgut probably caused the persistence of both the left and right embryonic hepatic arteries, and may have contributed to the presence of the hepatic artery on the posterior to the portal vein [18]. There have been no published reports on a vascular anomaly similar to our case in GC patients.

A point of caution during the surgery is the manipulation of the suprapancreatic lymph node dissection. In our department, we use a preemptive retropancreatic approach to conduct suprapancreatic lymph node dissection more safely [4,8]. This method is performed based on the following concepts that the release of the adherence between the retroperitoneum surface and retropancreatic fascia is a crucial step during the retropancreatic space dissection. Lifting the mesogastrium, including the suprapancreatic lymph nodes, forward provides an excellent operative field and prevents contact with the pancreas during suprapancreatic lymph node dissection [4]. This technique was also used this time to approach the suprapancreatic lymph node dissection. In this case, anatomic displacement was observed, and attention should be paid to PV injury at the time of lymph node no.8 and 12 dissection. First, the anterior surface of the PV and the nerve plexus around the CHA were identified, and the PV were secured with vascular tape after dissecting the entire circumference. By pulling the vascular tape gently to the ventral side, lymph node dissection no.8 and 12 of the dorsal side of the PV could be performed safely. Careful manipulation of the anatomic abnormalities identified on the preoperative images may prevent unintentional bleeding.

Furthermore, the right gastric artery branched from the proper hepatic artery and ran along the ventral side of the PV, the left gastric vein passed in front of the CHA and flowed into the PV, and the right gastric vein flowed into the splenic vein in this case. As all of these vessels were located in close proximity to the PV, careless use of forceps could lead to bleeding. Therefore, it is important to estimate the location of the vascular ligation based on preoperative imaging, and ultimately confirm it based on intraoperative findings, to avoid blind procedure.

Also, the present case was initially diagnosed as unresectable GC and underwent conversion surgery after chemotherapy was initiated, after which the cancer was evaluated to be resectable.

Chemotherapy is indicated as first-line treatment for unresectable GC [19,20]. With advances in chemotherapy, including anticancer and molecular-targeted drugs, conversion surgery has become increasingly valuable, and improvements in long-term prognosis have been reported. However, there is no consensus regarding conversion surgery for GC. An International Retrospective Cohort Study of Conversion Therapy for Stage IV Gastric Cancer 1 (CONVO-GC-1 study) showed a possible survival benefit in patients who achieved R0 by surgery [21]. There are few reports of Minimally Invasive Surgery (robot-assisted surgery, laparoscopic surgery) as conversion surgery, especially robot-assisted approaches [22,23]. In conversion surgery, bulky lymph node dissection is required in many cases, and robot-assisted surgery enables precise lymphatic dissection with 3D images and anti-shake articulated forceps, with possibly fewer postoperative complications than conventional laparoscopic surgery [24].

Finally, although the fact that only one case was reported is a limitation, it will be important to accumulate rare vascular cases in the future and share the points to be cautious during the surgery.

## 4 Conclusions

We performed RTGD2 as a conversion surgery for a case of GC with the CHA passing behind the PV, which is relatively rare. In the robot-assisted gastrectomy is becoming common, it is also feasible for performing lymphadenectomy safely in conversion surgery with vascular anomalies.

## Ethical approval

The case report is exempt from ethical approval at authors' institution. It is only necessary to obtain the patient's consent.

## Funding

The authors declare that no funds, grants, or other support were received during the preparation of this manuscript.

## CRediT authorship contribution statement

All authors designed the project. Yuto Sakurai, and Yuma Ebihara processed the clinical data, drafted the manuscript, and created the figures. All authors interpreted the results, prepared the manuscript, and approved the final version of the manuscript.

## Guarantor

Yuto Sakurai, Yuma Ebihara.

## Patient consent

Written informed consent was obtained from the patient for publication of this case report and accompanying images. A copy of the written consent is available for review by the Editor-in-Chief of this journal on request.

## Declaration of competing interest

The authors declare no conflicts of interest.
